# Supplementary material for: A memory-driven auditory program ensures selective and precise vocal imitation in zebra finches
Source: Commun Biol. 2021 Sep 13;4:1065. doi: 10.1038/s42003-021-02601-4 (PMC8437935; doi:10.1038/s42003-021-02601-4)
Supplement: Supplementary file 2 — Supplementary Information [file 42003_2021_2601_MOESM2_ESM.pdf]

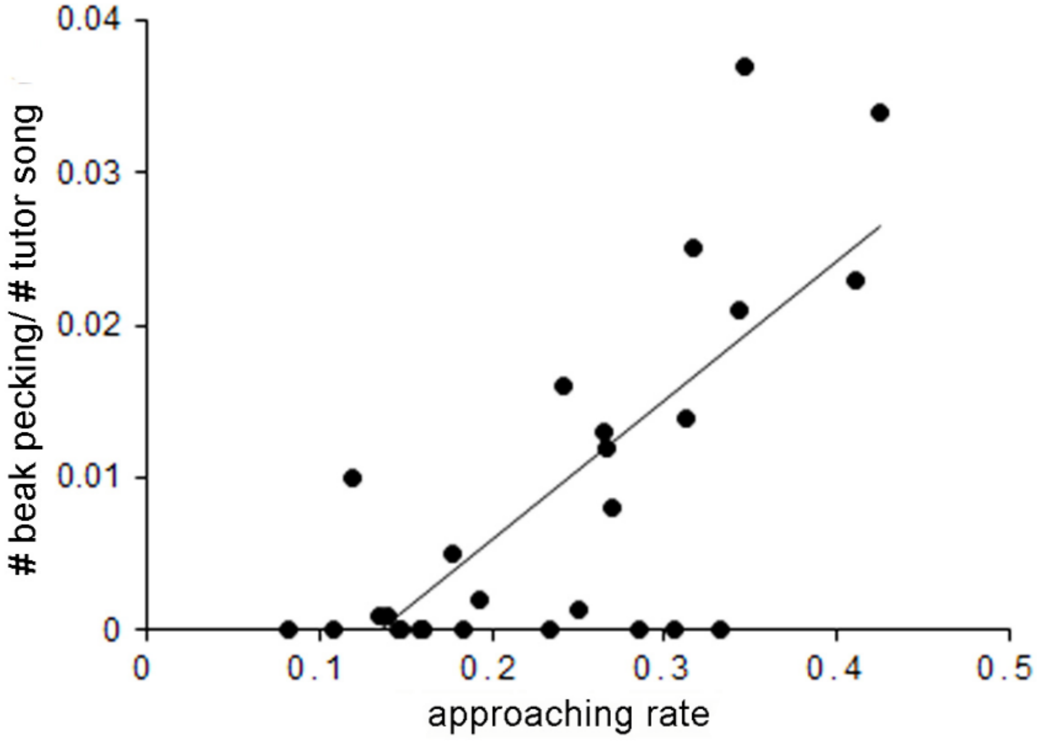

Supplementary Figure 1. The juveniles who had higher attentive approaching rate (i.e., number of approaching movement/ number of tutor song) were more likely to peck its tutor's beak immediately after approaching the tutor within 5 seconds (number of beak pecking/ number of tutor song; Pearson's Correlation, R^2=0.52, n=26 birds).

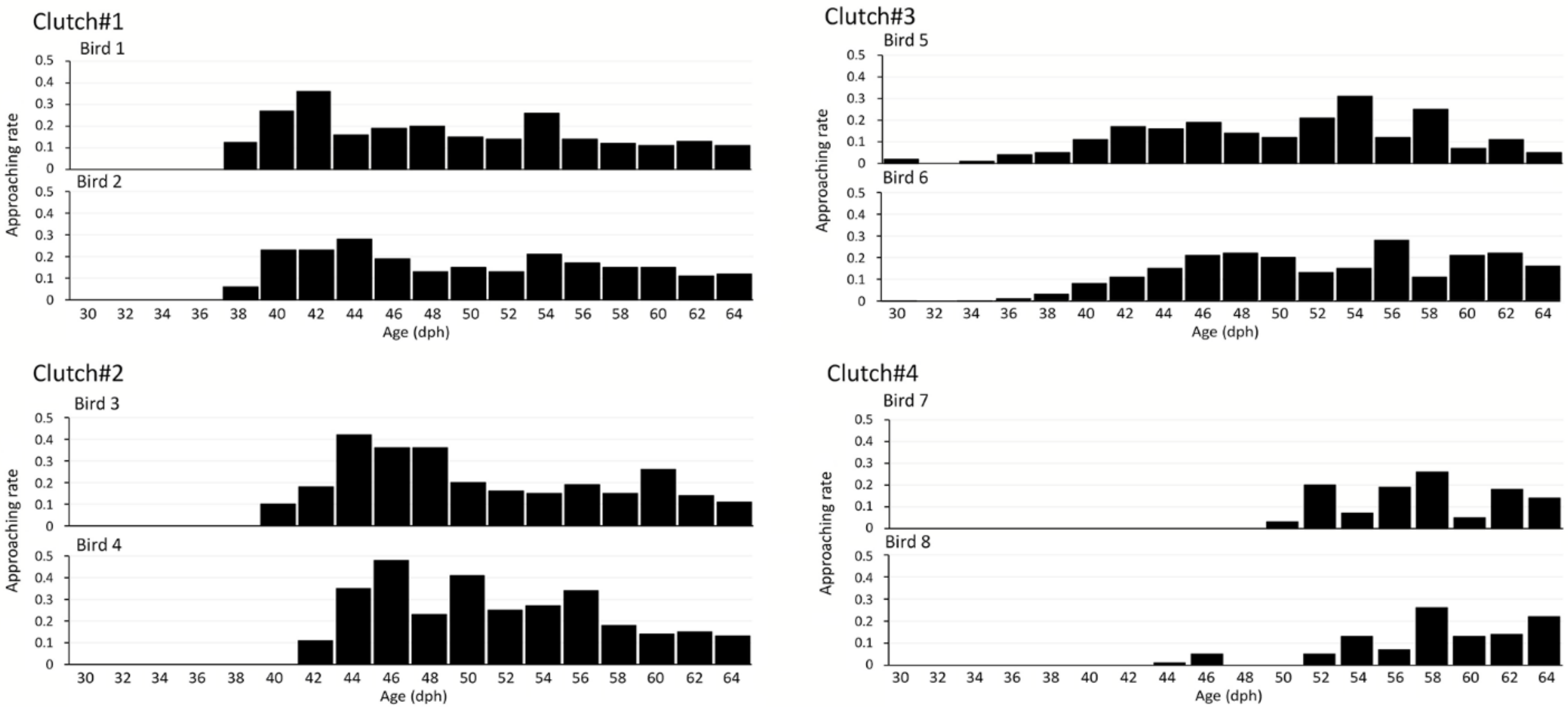

Supplementary Figure 2. Sibling effect on juveniles' approaching behavior. Two siblings from each of the four family clutches tended to develop a similar trajectory of attentive listening approaching behavior, as each pair of siblings from the same clutch had a similar onset and peak of approaching rate. Each panel represents one individual juvenile's attentive approaching trajectory across the sensitive period of vocal learning from 30-64 dph. The siblings from clutch #1 and #2 had higher approaching rate and much better song similarity match to the tutor than the siblings from clutch #3 and clutch #4, where both groups of juveniles had lower approaching rate and imitated poorly from the tutor.

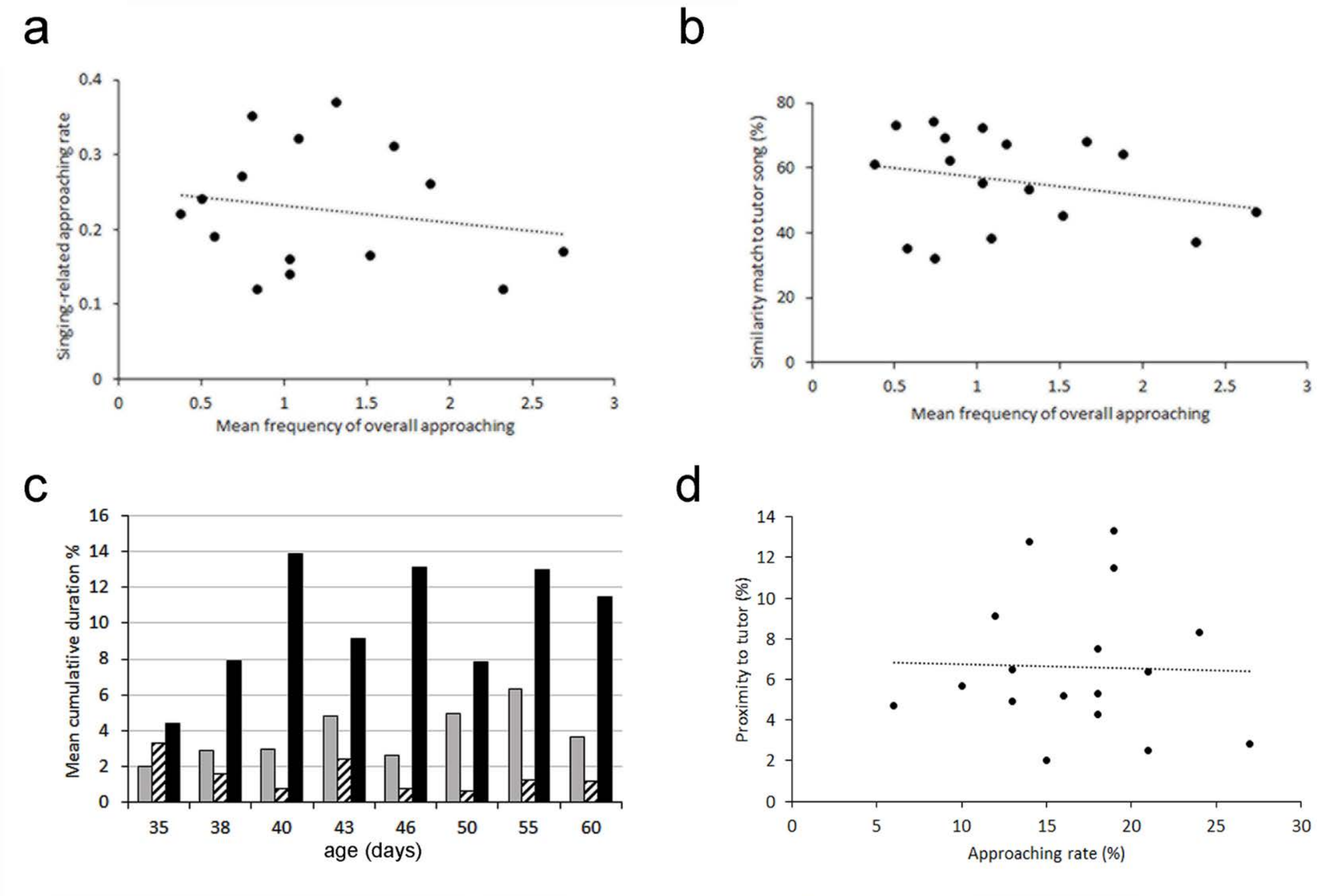

Supplementary Figure 3. Singing-induced listening/approaching rate did not correlate with the overall approaching rate or proximity between an approaching juvenile and its tutor. **a)** The overall approaching rate (i.e., mean frequency of approaching) between a juvenile and its tutor under all conditions did not correlate with the singing-induced listening/approaching behavior (Pearson's correlation, R^2=0.03). The overall approaching frequency was measured by the average number of approaches from an approaching juvenile to its tutor within 5 cm and under all social conditions per 6 minute recording. **b)** the overall approaching rate (mean frequency) under all social conditions did not correlate with similarity match to the tutor song. Each dot represents one juvenile bird at the age of 46-53 dph (R^2=0.06, n=17 birds). **c)** An example shows that three siblings from the same clutch had different social bonding with their rearing tutors (measured by the cumulative duration of staying together within 5 cm distance between an approaching juvenile and its tutor). One of the three siblings (black bar) consistently stayed closer to their adult tutor during the sensitive period, and yet the other sibling (i.e., gray bar) had the best tutor song imitation; **d)** Proximity between an approaching juvenile and its tutor did not correlate with "tutor singing-induced" approaching rate (number of approaches/number of tutor song; R^2=0.02). Proximity was measured as the percentage of mean cumulative duration of two birds staying together < 5cm per 6 minute video recording.

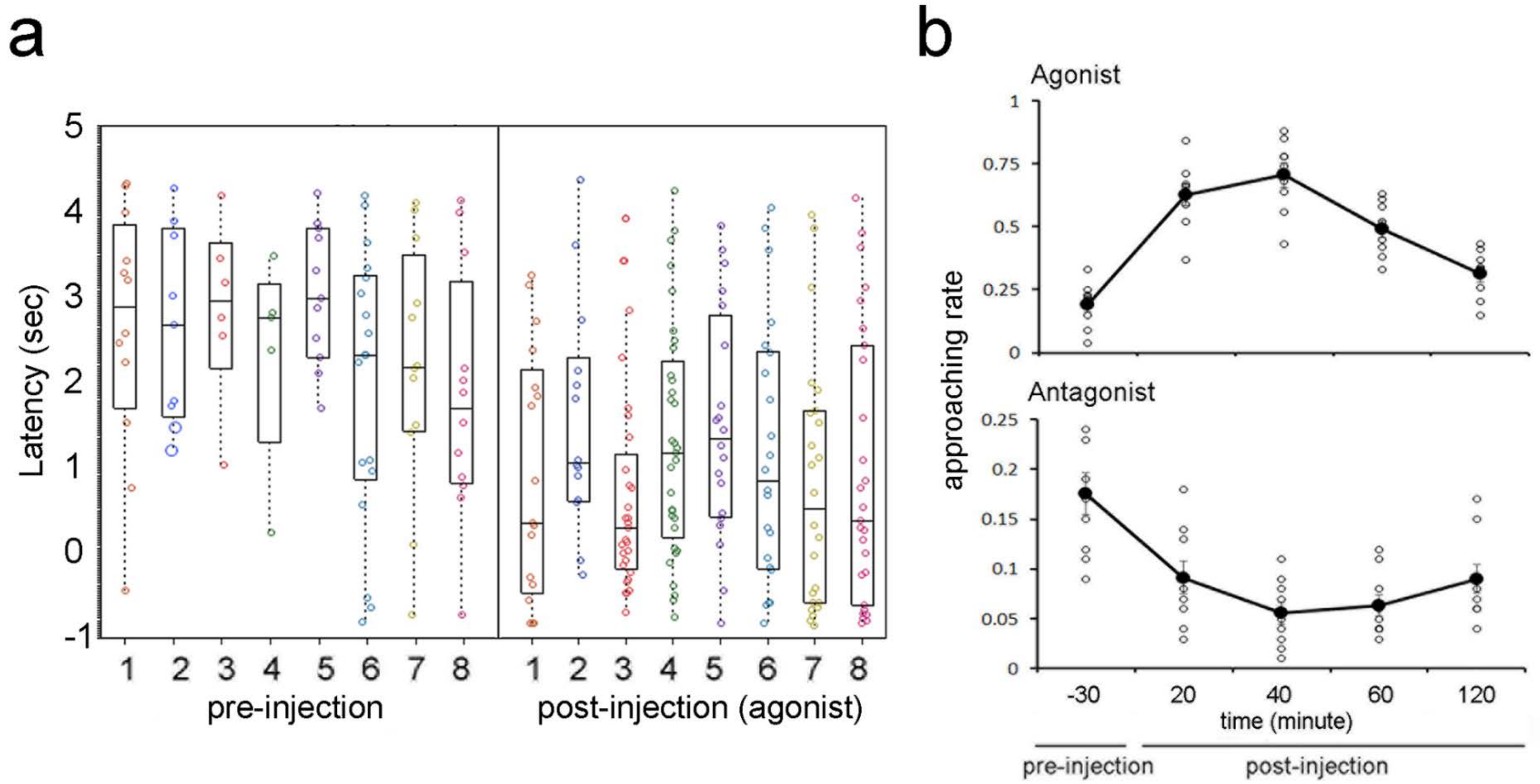

Supplementary Figure 4. a) Individual differences in the time latency (seconds) of approaching behavior before and after injection of dopamine agonist (nomifensine). Each colored box plot depicts the latency of approaching behavior of each of eight juveniles. In each boxplot, the lines from the top to bottom were defined as the third quartile, median, and first quartile. **b)** The effect of subcutaneous administration of dopamine agonist (top panel, n=9 birds) and antagonist SCH23390 (lower panel, n=9 birds) on the approaching behavior before (30 minutes) and after injection (over the period of two hours post-injection). The graph shows the mean and standard error of the approaching rate.

Supplementary Table 1. The number of animals used in each experiment.

Experiment	Sample size
Exp. 1. Quantification of listening/approaching behavior (recorded 30-65 days old).	33 juvenile males, 8 juvenile females
Quantification of beak pecking behavior.	26 juvenile males (birds from Exp.1)
Quantification of vocal silence	12 juvenile males (birds from Exp.1)
Quantification of latency of approaching	10 juvenile males (birds from Exp.1)
Exp. 2. Quantification of social proximity and overall approaching behavior.	17 juvenile males (birds from Exp.1)
Exp. 3 The effect of song playback on approaching behavior	6 juvenile males
Exp. 4. Tutor removal (removal between 35-45 days old) and re-exposure at 46 days old.	8 juvenile males
Exp. 5. Presentation of tutor or stranger adults after exposure of tutor song by 45 days.	8 juvenile males
Exp. 6. Playback of tutor or stranger song after exposure of tutor song by 45 days old.	8 juvenile males
Exp. 7. Father tutored after 46 days old.	8 juvenile males
Exp. 8. Correlation of song imitation and approaching behavior	23 juveniles (birds from Exp.1)
Exp. 9. Dopamine agonist (agonist vs. control)	17 juveniles (9 agonist and 8 control birds)
Exp. 10. Dopamine antagonist (antagonist vs. control)	18 juveniles (9 antagonist and 9 control birds)
Exp. 11. Gene expression (IEG) study	31 juveniles
