# Supplementary material for: A memory-driven auditory program ensures selective and precise vocal imitation in zebra finches
Source: Commun Biol. 2021 Sep 13;4:1065. doi: 10.1038/s42003-021-02601-4 (PMC8437935; doi:10.1038/s42003-021-02601-4)
Supplement: Supplementary file 3 — Description of Additional Supplementary Files [file 42003_2021_2601_MOESM3_ESM.pdf]

Description of Additional Supplementary Files

File name: Supplementary Movie 1

Description: Listening/approaching behavior of three juvenile zebra finches. Three juvenile males at 55 dph (each bird marked with blue, green, and yellow paints respectively) produced plastic song at the beginning of the video recording (07:39:00-07:39:21). As soon as the father tutor (marked by Magenta) started singing, all juveniles stopped singing, feeding and other ongoing behaviors, and two of them (blue and yellow birds) immediately oriented and approached their father tutor.

File name: Supplementary Movie 2

Description: Juveniles (painted with yellow and blue) immediately approached their tutor after the onset of tutor singing. After approaching, one of the juveniles (i.e., the bird with yellow paint) also pecked the beak of its father tutor.

File name: Supplementary Movie 3

Description: Juveniles (painted with yellow and blue) immediately approached their tutor (marked by magenta) even before the onset of tutor singing (10:33:15). The juveniles probably attentively observed a subtle gesture movement before the onset of tutor song.

File name: Supplementary Movie 4

Description: A father tutor (marked by Magenta) sang to one of his juveniles (painted with blue) who received subcutaneous injection of dopamine antagonist and had significantly reduced approaching to his father tutor. The other juvenile (i.e., the bird with green paint) was a saline-injection control and he approached the father after father's singing.

File name: Supplementary Data 1.

Description: Source data for Figures 1-4.

File name: Supplementary Data 2.

Description: Source data for Supplementary Figures 1-4.
